# Arthroscopic Repair of Rotator Cuff Tears Using a Suture Hook With Lasso Loop for Forward Suture Passing

**DOI:** 10.1016/j.eats.2023.10.010

**Published:** 2024-01-01

**Authors:** Wenbo Yang, Hong Wang, Zengu Shao, Wei Huang

**Affiliations:** Department of Orthopaedics, Union Hospital, Tongji Medical College, Huazhong University of Science and Technology, Wuhan, China

## Abstract

Rotator cuff injury is a common shoulder injury. Arthroscopic repair of rotator cuff tears has become a common way. Suture anchor is a commonly used fixation device. Threading the suture through the rotator cuff tendon is a key step in arthroscopic repair of rotator cuff tears. Common methods of passing suture through the rotator cuff tendon include using a suture hook or rotator cuff suture passer device. However, the suture using suture hook is a kind of reverse suture, and the operation is more troublesome. The rotator cuff suture passer device shows poor economy, which cannot be ignored at the risk of needle core breakage. We combined the advantages of the two suture passing methods to design an improved method by using a suture hook with the Lasso loop for forward suture passing. The key step of our improved technique is to deliver the end of the anchor suture 2-3 cm into the tip opening of suture hook, and ejecting the suture through pushing the Lasso loop after suture forward passing through the tendon. Our technique has achieved economy, safety, and simplicity, and has good clinical application value.

Rotator cuff injury is a common shoulder joint injury. Patients with rotator cuff tears may present with limited shoulder joint mobility and significant pain.^1^^,^^2^ Although nonsurgical intervention may be the initial recommendation for elderly patients who have a limited range of partial rotator cuff tears and minimal mobility requirements. For more patients, however, surgery is a better option. After undergoing surgery, patients typically experience pain relief, partial restoration of function, and partial recovery of local anatomy. Arthroscopic rotator cuff sutures are typically performed in the absence of significant local atrophy or when there is a need to remove substantial scarring, provided that the conditions of the local muscle and soft tissues allow for it. The rotator cuff is usually fixed and sutured with the suture anchor.^3^ The key step in rotator cuff suture surgery is to properly shuttle the suture of the anchor through the tendon and tie in place. In traditional procedures, suture hooks or a rotator cuff suture passer device can be used to thread the suture through the tendon.^4^^,^^5^ After the anchor is fixed, the suture of the anchor is located below the rotator cuff tendon, but the suture hook needs to be punctured from above the tendon. This is a "reverse" suturing, resulting in the operator having to frequently change the operating portal to fit the suturing position. In addition, when using a suture hook to suture the rotator cuff, the traction suture also need to be used. This increases the complexity of the procedure and also increases the risk of a stuck suture. In addition, in the traditional rotator cuff suture technique using suture hooks, the piercing direction is from the outside to the inside, which may damage the cartilage, increasing the risk of tissue damage. The use of the rotator cuff suture passer device allows the puncture to be made in a forward direction, and the location of the suture passing point can be more convenient and accurate. A non-negligible risk of the rotator cuff suture passer device is the breakage of the needle core at the head end.^6^ Once broken, the surgeon needs to spend a lot of effort to find and remove it, and it can cause additional harm to the patient. In addition, when using the rotator cuff suture passer device to sew a rotator cuff, the puncture point is limited to the distance from the tear edge, and the suture may be damaged during stitching. Therefore, the key to improve the efficiency of rotator cuff sutures under arthroscopy is to design an advanced scheme to make sutures of suture anchor pass through the tendon safely, conveniently, and accurately. Here, we designed an improved technique in which high-strength suture is loaded in the tip opening of the suture hook with pushing the Lasso loop for forward tendon suturing. Our technique combines the benefits of Lasso loop suture hook and rotator cuff passer device suture, eliminating the need for complex operations, while improving safety and economy.

## Surgical Technique

Our surgical protocol is shown in Video 1. The main steps are described as follows:

### Patient Preparation and Portal Establishment

**Fig 1:**
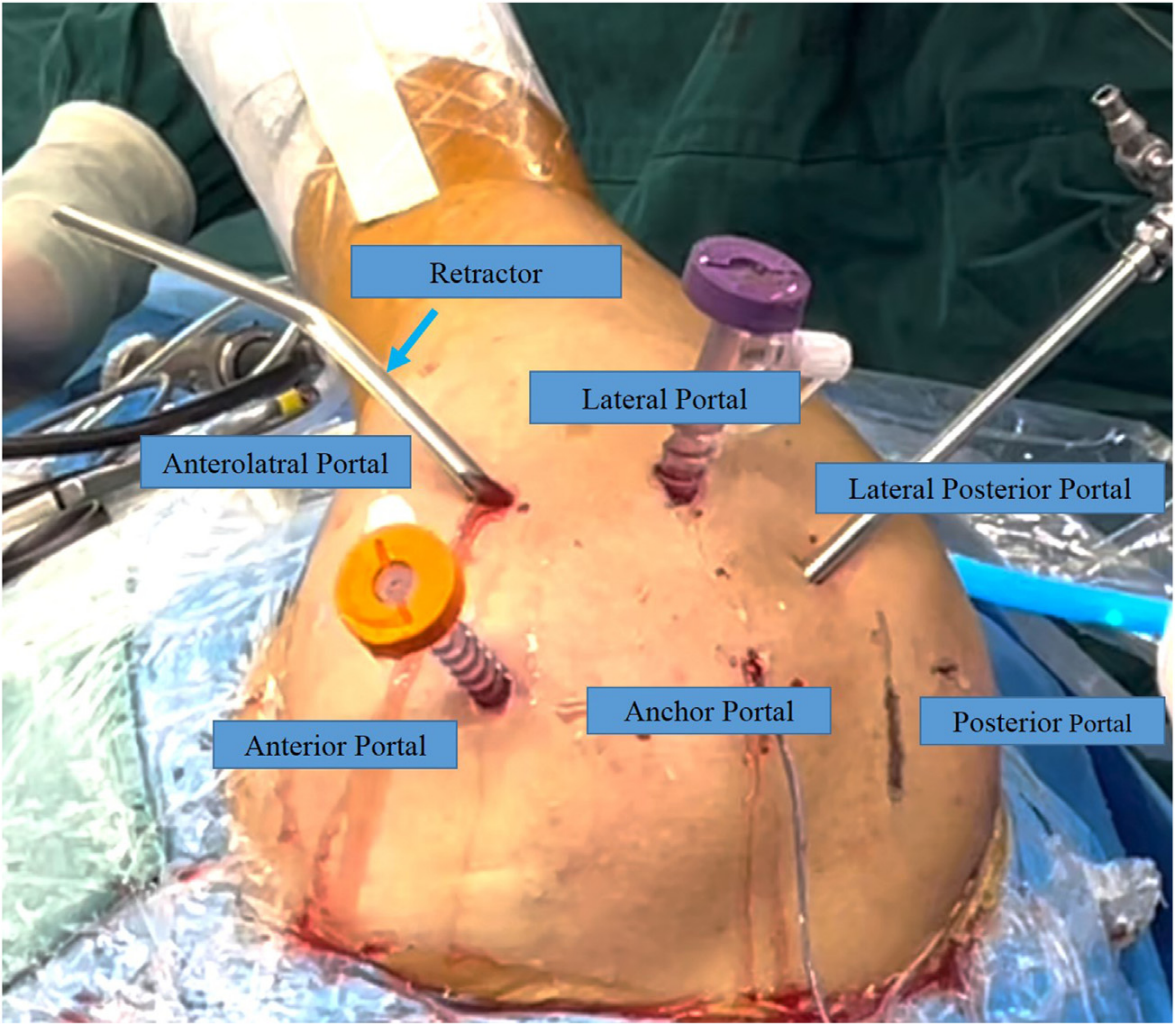
Location of the shoulder arthroscopic portal used in this example. This example is the rotator cuff injury in the right shoulder. The name of each portal is marked in the figure. The patient is placed in a lateral position. The blue arrow indicates the retractor.

Usually, patients undergo general anesthesia. After the patient is placed in a lateral position and fully exposed to the shoulder joint of the affected side, routine disinfection should be performed. We first set up the posterior portal as the observation portal and the anterior portal as the operation portal to explore the glenohumeral joint. Then the lateral posterior portal was established for subacromial lesion observation. After the lateral portal is established (Fig 1), the lateral acromial sac is cleaned. Carefully investigate the internal structure of the joint to identify the site of injury.

### Tissue Freshening and Suture Anchor Implantation

**Fig 2:**
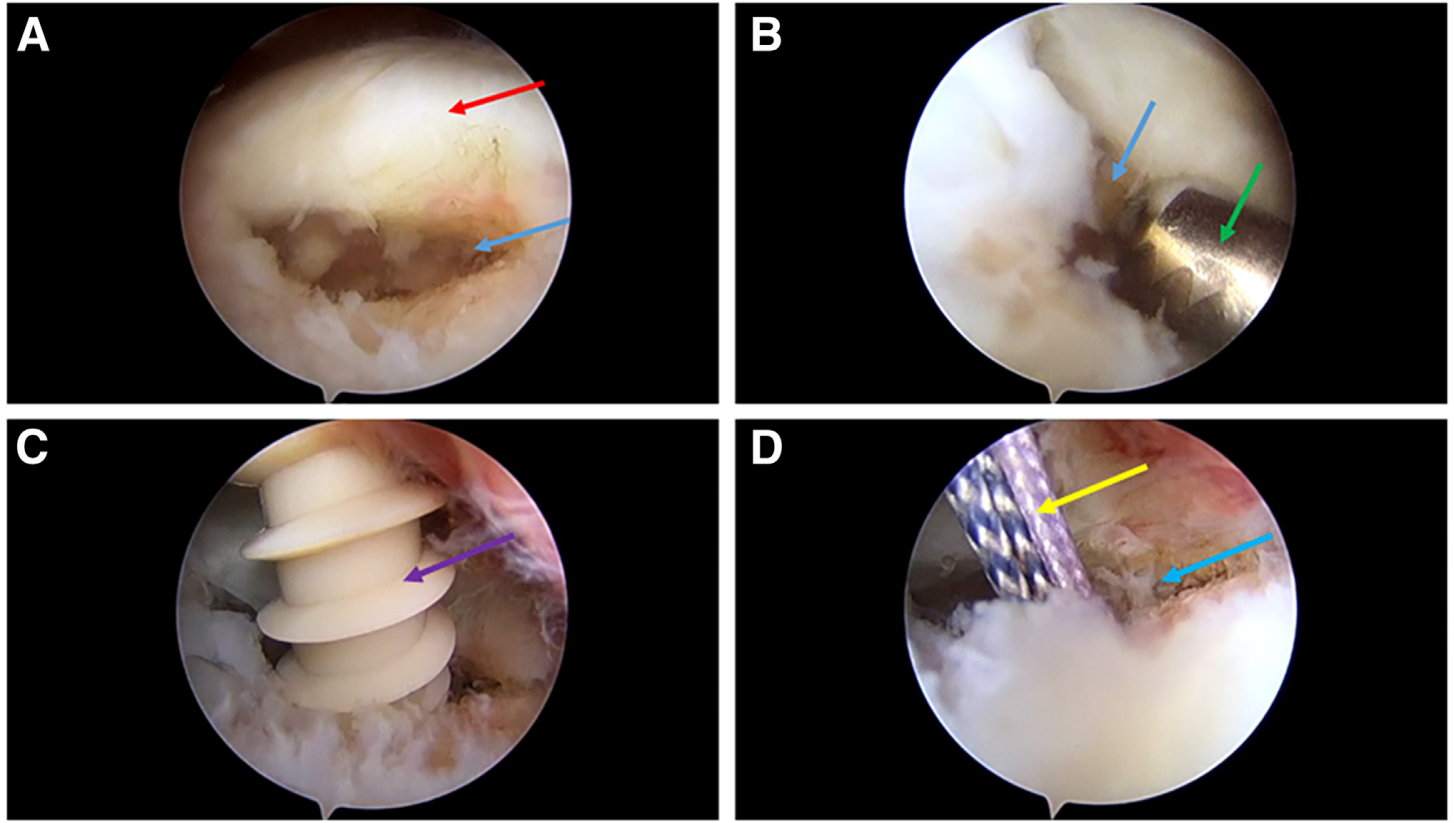
Tear site exposure, freshening, and suture anchor implantation. In this example, a small full layer tear is visible at the right shoulder supraspinatus muscle insertion through the lateral posterior observation portal. (A) Small full-layer rotator cuff tear. The lateral posterior portal acts as the observation portal. The red arrow indicates the supraspinatus tendon. The blue arrow indicates the location of the defect. The same as below in this figure. (B) The lesion was freshened by shaver. The lateral posterior portal acts as the observation portal. The lateral portal acts as the operation portal. The green arrow indicates the shaver. (C) Suture anchor is implanted. The purple arrow indicates the suture anchor. The lateral posterior portal is used as an observation portal. (D) The state of the implanted anchor. The yellow arrow indicates the double sutures loaded in the anchor.

The small full-layer tear of the rotator cuff is observed through the lateral posterior portal (Fig 2A), and the rotator cuff and foot print area are freshened (Fig 2B). The anchor portal was constructed under arthroscopic surveillance for implant of the suture anchor. A suture anchor (HEALIX PEEK Anchor w/ORTHOCORD; Depuy Mitek, Raynham, MA) was implanted at the center of the foot print area in preparation for single-row suture (Fig 2, C and D).

### Rotator Cuff Tendon Forward Suture Passing

**Fig 3:**
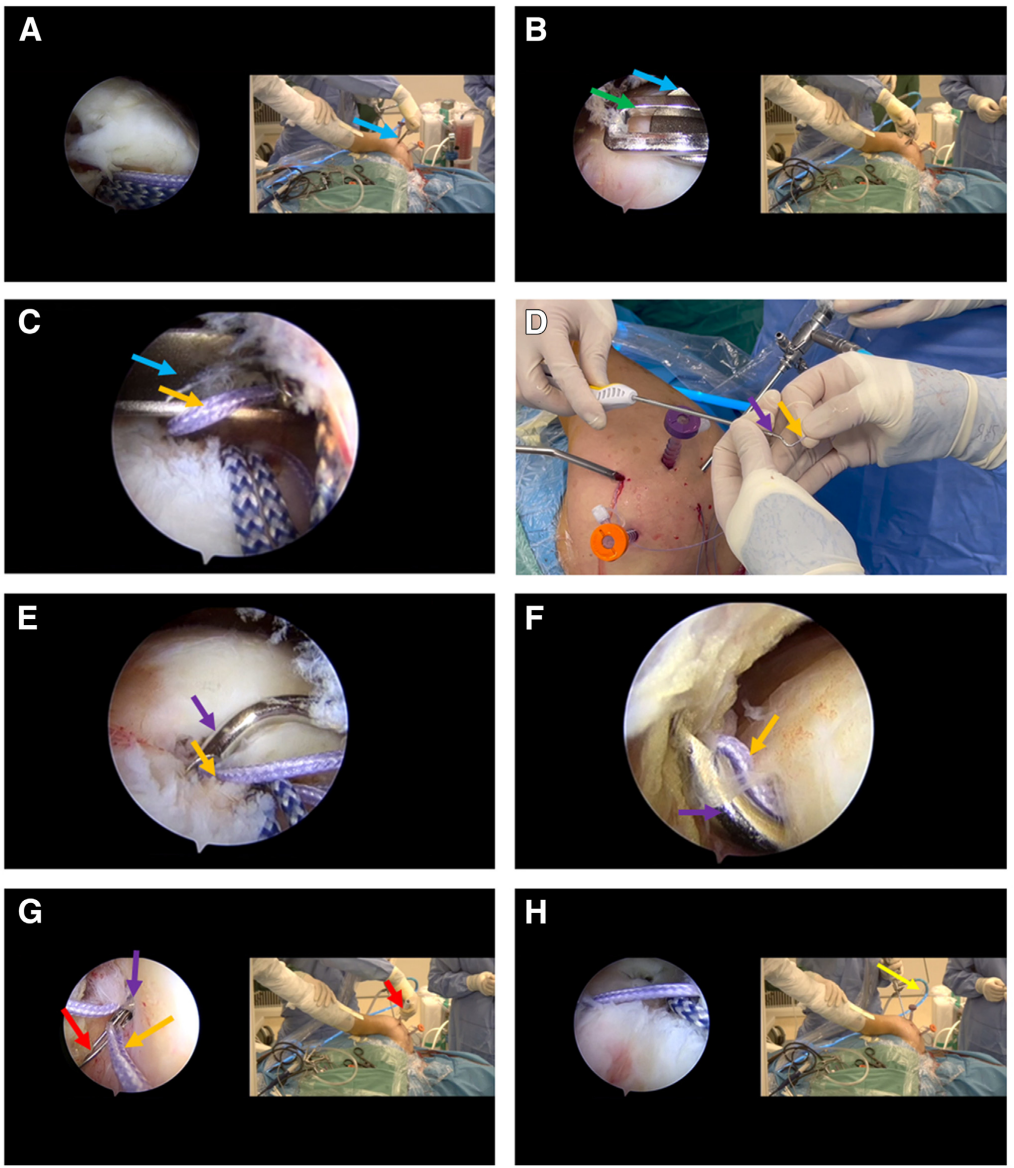
The process of the forward suture passing through the tendon (the first suture procedure). The lateral posterior portal is used as an observation portal. (A) The retractor is placed in the anterior lateral operation portal. The blue arrow indicates the retractor, as shown below in this figure. (B and C) The surgeon uses a grasper to pull out a suture. The green arrow indicates grasper, and the orange arrows indicate the suture that need to be passed through the tendon in this process, as shown below in this figure. (D) Suture hook loaded with anchor suture. Deliver the end of the anchor suture 2-3cm into the tip opening of suture hook. The purple arrow indicates the suture hook. (E) The suture hook reaches the injury site and is ready to be penetrated beneath the supraspinatus tendon. (F) The suture hook penetrates the rotator cuff tendon from the joint side to the bursa side. (G) Passing the end of suture out of the suture hook by pushing the Lasso loop. The red arrow indicates the Lasso loop. (H) Complete the first time of suture by pulling out the suture with the grasper. The yellow arrow indicates the pulled-out suture. Suture is pulled out through the lateral portal.

Prior to preparing the rotator cuff tendon for suturing, we place a retractor (Mitek Surgical Malleable Graft Retractor with Meniscal Deployment Gun; Depuy Mitek) in the anterior lateral operation portal (Figs 1 and 3A). This portal is used for fixation of the long biceps tendon in this patient. Anterior lateral operation portal and lateral operation portal can be combined into a single portal in this technique, that is, a separate portal newly established in the middle of the entrances of the two portals. The function of the retractor is to retract the soft tissue and form a channel to prevent the incarceration of the suture hook sutures. We deliver the grasper through the retractor into the joint cavity, pulled out a suture out of the joint for forward suture passing (Fig 3, B and C). We used a 45° (left) suture hook (Ideal Suture Shuttle, 45° Left; Depuy Mitek) loaded with a metal Lasso loop (Chia Percpasser Suture Passers, Depuy Mitek) for the suture. Unlike the norm, the metal Lasso loop needs to be moved backward rather than at the front exit of the suture hook. The Lasso loop is ∼2-3 cm away from the exit of the front end of the suture hook. Deliver the suture about 2-3 cm into the tip opening end of the suture hook (Fig 3D). The suture is in contact with the Lasso loop in the suture hook. The surgeon then places the suture hook loaded with suture end into the joint along the retractor via the anterior lateral operation portal. The posterior lateral part of the tear rotator cuff is subsequently forward sutured, that the suture hook pierces from the joint side of the tendon to the bursa side (Fig 3, E and F). After the suture hook pass through the tendon, push the metal lasso out of the suture hook, thereby squeezing out the suture (Fig 3G). If necessary, the grasper can be used to csecurely assist the operation of the suture hook. Finally, the suture hook is withdrawn, and the suture that is pushed out through the rotator cuff tendon tissue is pulled out through the lateral portal to complete a forward suture procedure (Fig 3H). Subsequently, another suture passing procedure is performed, similar to the previous ones. The posterior portal could be placed with a retractor, as needed.

**Fig 4:**
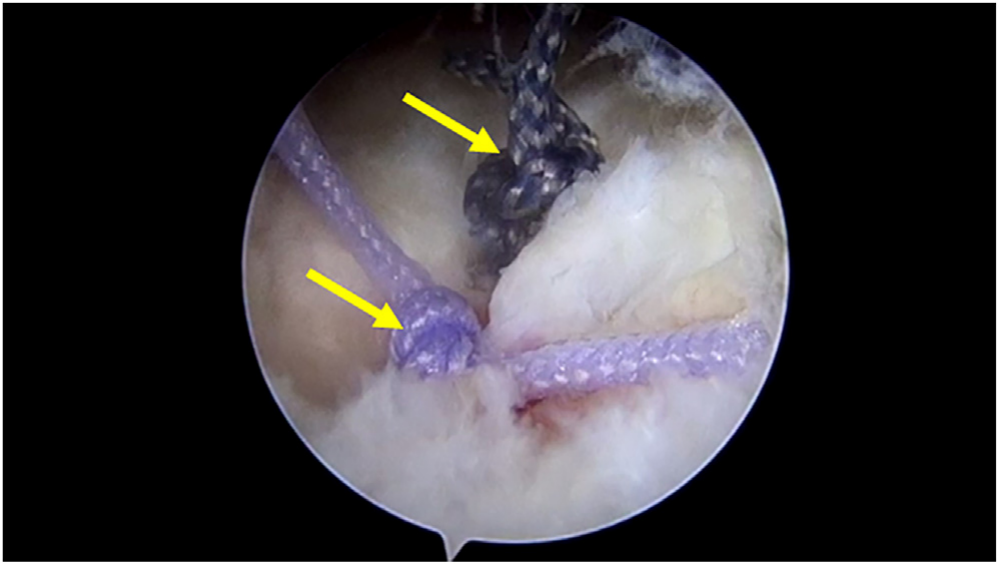
Complete 2 times forward suture passing and knot to finish single-row repair. The yellow arrows indicate two knots. Lateral posterior portal is observation portal.

After two suture passing has been completed, the knot fixing is completed though the lateral portal (Fig 4). Then we complete the suture surgery as usual.

## Discussion

Rotator cuff tears are common shoulder injuries that often require surgery. Traditional open surgery for rotator cuff repair has been replaced by mature arthroscopic techniques. During rotator cuff repair surgery, properly passing the suture through the tendon and tying is a key surgical step. The suture hook and rotator cuff passer device are the two most common devices used to pass suture through a tendon in arthroscopic surgery today. However, the traditional way of passing the suture with a suture hook is to take out the suture through the loop of the suture hook. Each threading must go through three steps in the shoulder joint: “push out the suture loop”, “pass the suture through the loop”, “pull out the suture hook with suture”, which is very fussy. Also, the suture hook needs to take the suture passing the tendon in the reverse direction, which means the puncture position may be inaccurate. In addition, the traditional suture hook method requires the surgeon to use a traction suture and change different portals to fit the suture at different locations, which makes the operation cumbersome. On the other hand, the rotator cuff suture passer device has the advantage of enabling forward suture passing, but it still has some risks or troubles, such as poor economy, needle core broken, etc. At the same time, the distance between the puncture point of the rotator cuff suture passer device and the tear edge is limited, which can easily result in insufficient suture strength in some cases. And this device is prone to damage the suture during puncture. In our clinical practice, we have found that the advantages of the two types of suture are complementary. Because of the variety of angles of the suture hook, when using the suture hook to suture the rotator cuff tendon, different types of hooks can be replaced, avoiding frequent changes in the operation portal and observation portal, and the selection of the suture site is more flexible. At the same time, the suture hook achieves better economy and avoids the risk of instrument damage. On the basis of advantages of the two options above, we combine them and propose an improved technique called forward-suture suture hook technique with automatic suture thread passing. In our improved technique, the idea of forward passing the suture through pushing the Lasso loop inside the suture hook is derived from the principle of the rotator cuff suture passer device, while retaining the advantage of the suture hook to repair the rotator cuff. It is worth mentioning that our improved technique can be used for continuous rotator cuff suture, which makes the repair of complex rotator cuff tears more convenient. The pearls and pitfalls of our technique are shown in Table 1, while the advantages and disadvantages are shown in Table 2. It is worth noting that our technique has the capability to reduce the number of portals and traction sutures in comparison to traditional techniques. Specifically, because of the diverse angle of suture hooks, there is a potential reduction of the required portals for operation. Our technique can be applied across various situations, whether using single-row anchors or double-row anchors. Furthermore, our technique effectively avoids cartilage damage due to the outward piercing direction. In the example that we report, we used a suture hook in the same direction to suture different parts of the broken cuff tendon through two different portals. In future clinical practice, this technique can also achieve direct forward suture through the same portal, using suture hooks in different directions. The clinician can also perform suture knotting directly through the lateral portal, which can further reduce the establishment of additional portals and further reduce the trauma of patients. At the same time, our technique is widely used, whether a single-row anchor suture or double-row anchor suture is suitable. In summary, our improved technique is expected to be an alternative to traditional suturing and become a common procedure for rotator cuff suturing.

**Table 1:** The Pearls and Pitfalls of the Forward-Suture Suture Hook Technique With Lasso Loop for Forward Suture Passing

Pearls	Pitfalls
1. We used a retractor for retracting the soft tissue to prevent the suture from getting stuck and falling out.	1. It is recommended that the physician do not use the cannulas when operating with the suture hook to avoid the slip of the suture.
2. The end of anchor suture is generally delivered into the tip opening of suture hook about 2-3 cm. If it is too short, it will fall out, and if it is too long, the suture will not convenient to be pushed out.	2. The suture must be totally pushed out by the Lasso loop before carefully exiting the suture hook to avoid taking the suture out.
3. Grasper can be used to assist the suture hook if it is difficult to penetrate the rotator cuff tendon.	
4. With this technique, only one suture hook with the appropriate angle and orientation is enough if we create more than one working portal. Conversely, only one working portal is enough if the suture hooks with different orientations (different angles) are used.	
5. The metal lasso loop used for pushing sutures can also be replaced with PDS	
6. The anterolateral approach and the lateral approach in this case can be used together as one approach.	

PDS, polydioxanone suture.

**Table 2:** The Advantages and Disadvantages of the Forward-Suture Suture Hook Technique With Lasso Loop for Forward Suture Passing

Advantages	Disadvantages
1. Our technique enables suture to be sutured forward without the use of traction sutures, which is fast and convenient.	1. The suture direction of our technique is opposite to that of the traditional suture hook suture process, which requires a learning process.
2. The portal for grasper to grab the suture is eliminated.	2. Once the suture loaded in the suture hook falls off before passing through the rotator cuff tendon, it needs to be reloaded again.
3. Our technique reduces the need to create additional operational portals, as suture hooks with different angels are available.	
4. Our technique is applicable to a wide range of tear types and suture techniques, such as single-row anchors, double-row anchors, etc.	
5. Our technique enables continuous suture and has a wide range of applications, which can be extended to related surgeries in the other joint.	
6. In our technique, the direction of suture hook puncture is forward. (Suture hook pierces from the joint side of the tendon to the bursa side), so it can effectively avoid cartilage damage caused by suture hook tip.	

## Disclosure

The authors report no conflicts of interest in the authorship and publication of this article. Full ICMJE author disclosure forms are available for this article online, as supplementary material.

## Supplementary Data

ICMJE author disclosure forms

Video 1 Introduction of the forward-suture suture hook technique with Lasso loop. In this video, we give a complete illustration of how we innovatively insert the suture into the tip opening end of the suture hook for forward suture, as well as the function of the retractor we creatively used.
